# 1-(4-Methyl-1-naphth­yl)ethanone

**DOI:** 10.1107/S1600536808035812

**Published:** 2008-11-13

**Authors:** Yong-Hong Hu, Xiao-Lei Zhao, Wen-Ge Yang, Jin-Feng Yao, Xiu-Tao Lu

**Affiliations:** a College of Life Science and Pharmaceutical Engineering, Nanjing University of Technolgy, Xinmofan Road No. 5 Nanjing, Nanjing 210009, People’s Republic of China

## Abstract

In the mol­ecule of the title compound, C_13_H_12_O, the two aromatic rings are oriented at a dihedral angle of 2.90 (3)°. An intra­molecular C—H⋯O hydrogen bond results in the formation of a non-planar six-membered ring, which adopts an envelope conformation. In the crystal structure, inter­molecular C—H⋯O hydrogen bonds link the mol­ecules.

## Related literature

For related structures, see: Dixon *et al.* (1981 ▶); Grummitt & Buck (1943 ▶); Merritt & Braun (1950 ▶). For bond-length data, see: Allen *et al.* (1987 ▶).
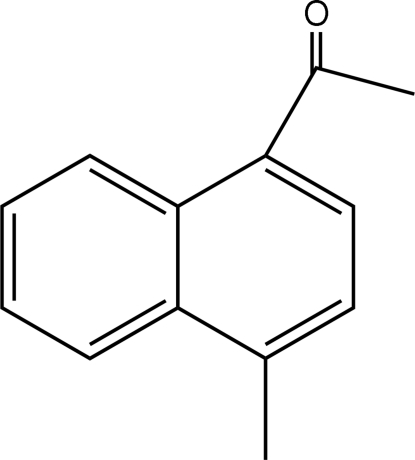


## Experimental

### 

#### Crystal data

C_13_H_12_O
*M* *_r_* = 184.23
Orthorhombic, 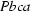

*a* = 15.449 (3) Å
*b* = 7.8290 (16) Å
*c* = 16.755 (3) Å
*V* = 2026.5 (7) Å^3^
*Z* = 8
Mo *K*α radiation
μ = 0.07 mm^−1^
*T* = 294 (2) K
0.30 × 0.20 × 0.10 mm

#### Data collection

Enraf–Nonius CAD-4 diffractometer
Absorption correction: ψ scan (North *et al.*, 1968 ▶) *T*_min_ = 0.978, *T*_max_ = 0.993
1932 measured reflections
1846 independent reflections
905 reflections with *I* > 2σ(*I*)
*R*_int_ = 0.000
3 standard reflections frequency: 120 min intensity decay: none

#### Refinement

*R*[*F* ^2^ > 2σ(*F* ^2^)] = 0.059
*wR*(*F* ^2^) = 0.155
*S* = 1.01
1846 reflections
127 parameters
H-atom parameters constrained
Δρ_max_ = 0.14 e Å^−3^
Δρ_min_ = −0.16 e Å^−3^

### 

Data collection: *CAD-4 Software* (Enraf–Nonius, 1989 ▶); cell refinement: *CAD-4 Software*; data reduction: *XCAD4* (Harms & Wocadlo, 1995 ▶); program(s) used to solve structure: *SHELXS97* (Sheldrick, 2008 ▶); program(s) used to refine structure: *SHELXL97* (Sheldrick, 2008 ▶); molecular graphics: *SHELXTL* (Sheldrick, 2008 ▶) and *PLATON* (Spek, 2003 ▶); software used to prepare material for publication: *SHELXTL*.

## Supplementary Material

Crystal structure: contains datablocks global, I. DOI: 10.1107/S1600536808035812/hk2564sup1.cif

Structure factors: contains datablocks I. DOI: 10.1107/S1600536808035812/hk2564Isup2.hkl

Additional supplementary materials:  crystallographic information; 3D view; checkCIF report

## Figures and Tables

**Table 1:** Hydrogen-bond geometry (Å, °)

*D*—H⋯*A*	*D*—H	H⋯*A*	*D*⋯*A*	*D*—H⋯*A*
C2—H2*A*⋯O	0.93	2.30	2.920 (4)	124
C13—H13*C*⋯O^i^	0.96	2.55	3.296 (4)	135

Symmetry code: (i) 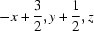.

**Fig. 1.**
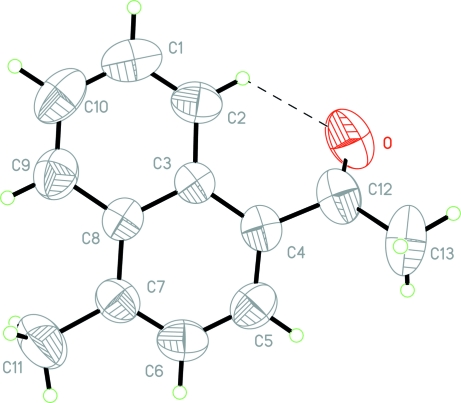
The molecular structure of the title molecule, with the atom- numbering scheme. Displacement ellipsoids are drawn at the 30% probability level. Hydrogen bond is shown as dashed line.

**Fig. 2.**
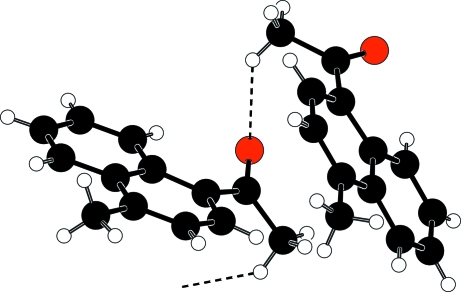
A partial packing diagram. Hydrogen bonds are shown as dashed lines.

## supplementary crystallographic information

### Comment

The title compound is a dye and plastic processing aid of intermediate, 1,4-naphthalenedicarboxylic acid. As part of our ongoing studies in this area, we report herein its crystal structure.

In the title compound (Fig. 1), the bond lengths (Allen *et al.*, 1987) and angles are within normal ranges. Rings A (C3-C8) and B (C1-C3/C8-C10) are, of course, planar and the dihedral angle between them is A/B = 2.90 (3)°. The intramolecular C-H···O hydrogen bond (Table 1) results in the formation of a nonplanar six-membered ring C (C2-C4/C12/O/H2A) adopting envelope conformation with O atom displaced by -0.533 (3) Å from the plane of the other ring atoms.

In the crystal structure, intermolecular C-H···O hydrogen bonds (Table 1) link the molecules (Fig. 2), in which they may be effective in the stabilization of the structure.

### Experimental

In a three-necked flask, naphthalene (256.0 g, 2.00 mol), paraformaldehyde (110.0 g, 1.22 mol), glacial acetic acid (260 ml), concentrated hydrochloric acid (362 ml) and phosphoric acid (165 ml, 85%) are heated with efficient stirring in a water bath at 353-358 K for 6 h. The product is washed two times with cold water (1 liter), a solution of potassium carbonate (20.0 g) in cold water (500 ml), and finally with cold water (500 ml). Ether (200 ml) is added to the oil layer and the solution is given a preliminary drying with anhydrous potassium carbonate (10.0 g) for 1 h. The lower aqueous layer is separated and the ether solution again dried with potassium carbonate (20.0 g) for 8-10 h. The ether solution is distilled first at atmospheric pressure to remove the ether, and then followed by distillation under reduced pressure to obtain 1-chloromethylnaphthalene. In a three-necked flask, magnesium (63.2 g), absolute ether (100 ml), a crystal of iodine, a solution of 1-chloromethylnaphthalene (150.0 g, 0.85 mol) in absolute ether (750 ml) and absolute ether (1080 ml) are mixed, and the ether solution of the chloride is added to the mixture in 5 h, and then stirred and heated at reflux for an additional 1 h to obtain 1-methylnaphthalene. The yield was 88-92% (Grummitt & Buck, 1943). Acetyl chloride (39.3 g, 0.48 mol, 38 ml) is added over 45 min to a stirred mixture of 1-methylnaphthalene (Aldrich) (67.0 g, 0.48 mol), dry dichloromethane (340 ml) and finely ground anhydrous aluminium chloride (76.0 g, 0.57 mol) at 273 K. After the addition is completed, the mixture is stirred at ambient temperature for 4 h, and then heated under reflux for 2.5 h (Dixon *et al.*, 1981). The reaction solution is washed with hydrochloric acid many times. The aqueous phase followed by distillation under reduced pressure gave the title compound (yield; 71%) (Merritt & Braun, 1950). Crystals suitable for X-ray analysis are obtained by slow evaporation of an petroleum ether solution.

### Refinement

H atoms were positioned geometrically, with C-H = 0.93 and 0.96 Å for aromatic and methyl H, respectively, and constrained to ride on their parent atoms with U_iso_(H) = xU_eq_(C), where x = 1.5 for methyl H and x = 1.2 for aromatic H atoms.

### Crystal data

**Table:**

C_13_H_12_O	*F*_000_ = 784
*M**_r_* = 184.23	*D*_x_ = 1.208 Mg m^−^^3^
Orthorhombic, *P**b**c**a*	Mo *K*α radiation λ = 0.71073 Å
Hall symbol: -P 2ac 2ab	Cell parameters from 25 reflections
*a* = 15.449 (3) Å	θ = 9–13º
*b* = 7.8290 (16) Å	µ = 0.08 mm^−^^1^
*c* = 16.755 (3) Å	*T* = 294 (2) K
*V* = 2026.5 (7) Å^3^	Block, colorless
*Z* = 8	0.30 × 0.20 × 0.10 mm

### Data collection

**Table:**

Enraf–Nonius CAD-4 diffractometer	*R*_int_ = 0.0000
Radiation source: fine-focus sealed tube	θ_max_ = 25.3º
Monochromator: graphite	θ_min_ = 2.4º
*T* = 294(2) K	*h* = 0→18
ω/2θ scans	*k* = 0→9
Absorption correction: ψ scan(North *et al.*, 1968)	*l* = 0→20
*T*_min_ = 0.978, *T*_max_ = 0.993	3 standard reflections
1932 measured reflections	every 120 min
1846 independent reflections	intensity decay: none
905 reflections with *I* > 2σ(*I*)	

### Refinement

**Table:**

Refinement on *F*^2^	Secondary atom site location: difference Fourier map
Least-squares matrix: full	Hydrogen site location: inferred from neighbouring sites
*R*[*F*^2^ > 2σ(*F*^2^)] = 0.059	H-atom parameters constrained
*wR*(*F*^2^) = 0.155	*w* = 1/[σ^2^(*F*_o_^2^) + (0.05*P*)^2^ + 0.8*P*] where *P* = (*F*_o_^2^ + 2*F*_c_^2^)/3
*S* = 1.01	(Δ/σ)_max_ < 0.001
1846 reflections	Δρ_max_ = 0.14 e Å^−^^3^
127 parameters	Δρ_min_ = −0.16 e Å^−^^3^
Primary atom site location: structure-invariant direct methods	Extinction correction: none

### Special details

**Table:**

Geometry. All e.s.d.'s (except the e.s.d. in the dihedral angle between two l.s. planes) are estimated using the full covariance matrix. The cell e.s.d.'s are taken into account individually in the estimation of e.s.d.'s in distances, angles and torsion angles; correlations between e.s.d.'s in cell parameters are only used when they are defined by crystal symmetry. An approximate (isotropic) treatment of cell e.s.d.'s is used for estimating e.s.d.'s involving l.s. planes.
Refinement. Refinement of *F*^2^ against ALL reflections. The weighted *R*-factor *wR* and goodness of fit *S* are based on *F*^2^, conventional *R*-factors *R* are based on *F*, with *F* set to zero for negative *F*^2^. The threshold expression of *F*^2^ > σ(*F*^2^) is used only for calculating *R*-factors(gt) *etc*. and is not relevant to the choice of reflections for refinement. *R*-factors based on *F*^2^ are statistically about twice as large as those based on *F*, and *R*- factors based on ALL data will be even larger.

### Fractional atomic coordinates and isotropic or equivalent isotropic displacement parameters (Å^2^)

**Table:**

	*x*	*y*	*z*	*U*_iso_*/*U*_eq_	
O	0.72633 (18)	−0.0605 (3)	0.52877 (14)	0.1022 (9)	
C1	0.4927 (2)	0.1819 (5)	0.5918 (2)	0.0830 (11)	
H1A	0.4590	0.1827	0.5457	0.100*	
C2	0.5750 (2)	0.1221 (4)	0.58895 (18)	0.0665 (9)	
H2A	0.5973	0.0829	0.5407	0.080*	
C3	0.62766 (19)	0.1181 (3)	0.65824 (17)	0.0498 (7)	
C4	0.7163 (2)	0.0658 (3)	0.65707 (18)	0.0573 (8)	
C5	0.7619 (2)	0.0651 (4)	0.7276 (2)	0.0679 (9)	
H5A	0.8198	0.0326	0.7272	0.082*	
C6	0.7234 (2)	0.1121 (4)	0.79985 (19)	0.0710 (10)	
H6A	0.7554	0.1034	0.8467	0.085*	
C7	0.6410 (2)	0.1698 (4)	0.80342 (17)	0.0611 (8)	
C8	0.59134 (18)	0.1753 (3)	0.73121 (18)	0.0531 (7)	
C9	0.5063 (2)	0.2390 (5)	0.7314 (2)	0.0731 (10)	
H9A	0.4825	0.2794	0.7787	0.088*	
C10	0.4583 (2)	0.2424 (4)	0.6633 (3)	0.0829 (11)	
H10A	0.4022	0.2854	0.6645	0.100*	
C11	0.6043 (3)	0.2281 (5)	0.88156 (17)	0.0941 (13)	
H11A	0.6474	0.2163	0.9225	0.141*	
H11B	0.5548	0.1597	0.8948	0.141*	
H11C	0.5873	0.3457	0.8775	0.141*	
C12	0.7628 (3)	0.0157 (4)	0.5827 (2)	0.0733 (10)	
C13	0.8571 (2)	0.0651 (4)	0.5755 (2)	0.0961 (13)	
H13A	0.8794	0.0259	0.5253	0.144*	
H13B	0.8893	0.0137	0.6183	0.144*	
H13C	0.8625	0.1871	0.5786	0.144*	

### Atomic displacement parameters (Å^2^)

**Table:**

	*U*^11^	*U*^22^	*U*^33^	*U*^12^	*U*^13^	*U*^23^
O	0.127 (2)	0.098 (2)	0.0814 (16)	−0.0038 (18)	0.0255 (17)	−0.0239 (15)
C1	0.081 (3)	0.079 (3)	0.090 (3)	−0.011 (2)	−0.028 (2)	0.017 (2)
C2	0.078 (2)	0.061 (2)	0.061 (2)	−0.0079 (19)	−0.0101 (18)	0.0077 (16)
C3	0.0557 (18)	0.0421 (16)	0.0516 (16)	−0.0036 (14)	0.0025 (15)	0.0035 (14)
C4	0.066 (2)	0.0455 (17)	0.0601 (18)	−0.0005 (16)	0.0093 (18)	0.0039 (15)
C5	0.063 (2)	0.060 (2)	0.081 (2)	0.0066 (17)	−0.005 (2)	0.0095 (18)
C6	0.079 (3)	0.074 (2)	0.061 (2)	0.000 (2)	−0.0125 (18)	0.0105 (17)
C7	0.073 (2)	0.0565 (19)	0.0535 (18)	−0.0008 (18)	0.0024 (18)	0.0037 (15)
C8	0.0516 (18)	0.0464 (16)	0.0614 (18)	−0.0009 (15)	0.0018 (16)	0.0054 (14)
C9	0.066 (2)	0.074 (2)	0.079 (2)	0.005 (2)	0.010 (2)	0.0109 (19)
C10	0.053 (2)	0.072 (2)	0.124 (3)	−0.0011 (19)	0.000 (2)	0.021 (2)
C11	0.122 (3)	0.101 (3)	0.059 (2)	0.013 (3)	0.011 (2)	−0.004 (2)
C12	0.095 (3)	0.0495 (19)	0.076 (2)	0.0060 (19)	0.021 (2)	0.0012 (18)
C13	0.079 (3)	0.081 (3)	0.128 (3)	0.007 (2)	0.046 (2)	0.004 (2)

### Geometric parameters (Å, °)

**Table:**

O—C12	1.220 (4)	C7—C8	1.434 (4)
C1—C2	1.357 (4)	C7—C11	1.498 (4)
C1—C10	1.394 (5)	C8—C9	1.405 (4)
C1—H1A	0.9300	C9—C10	1.360 (4)
C2—C3	1.418 (4)	C9—H9A	0.9300
C2—H2A	0.9300	C10—H10A	0.9300
C3—C8	1.418 (4)	C11—H11A	0.9600
C3—C4	1.430 (4)	C11—H11B	0.9600
C4—C5	1.376 (4)	C11—H11C	0.9600
C4—C12	1.491 (4)	C12—C13	1.513 (5)
C5—C6	1.398 (4)	C13—H13A	0.9600
C5—H5A	0.9300	C13—H13B	0.9600
C6—C7	1.352 (4)	C13—H13C	0.9600
C6—H6A	0.9300		
			
C2—C1—C10	120.3 (3)	C3—C8—C7	120.4 (3)
C2—C1—H1A	119.9	C10—C9—C8	121.0 (3)
C10—C1—H1A	119.9	C10—C9—H9A	119.5
C1—C2—C3	121.1 (3)	C8—C9—H9A	119.5
C1—C2—H2A	119.4	C9—C10—C1	120.5 (3)
C3—C2—H2A	119.4	C9—C10—H10A	119.8
C8—C3—C2	118.2 (3)	C1—C10—H10A	119.8
C8—C3—C4	118.8 (3)	C7—C11—H11A	109.5
C2—C3—C4	123.0 (3)	C7—C11—H11B	109.5
C5—C4—C3	118.7 (3)	H11A—C11—H11B	109.5
C5—C4—C12	118.1 (3)	C7—C11—H11C	109.5
C3—C4—C12	123.2 (3)	H11A—C11—H11C	109.5
C4—C5—C6	121.7 (3)	H11B—C11—H11C	109.5
C4—C5—H5A	119.2	O—C12—C4	121.7 (3)
C6—C5—H5A	119.2	O—C12—C13	120.8 (3)
C7—C6—C5	121.8 (3)	C4—C12—C13	117.5 (3)
C7—C6—H6A	119.1	C12—C13—H13A	109.5
C5—C6—H6A	119.1	C12—C13—H13B	109.5
C6—C7—C8	118.5 (3)	H13A—C13—H13B	109.5
C6—C7—C11	119.8 (3)	C12—C13—H13C	109.5
C8—C7—C11	121.7 (3)	H13A—C13—H13C	109.5
C9—C8—C3	118.9 (3)	H13B—C13—H13C	109.5
C9—C8—C7	120.6 (3)		
			
C10—C1—C2—C3	0.5 (5)	C2—C3—C8—C7	178.4 (3)
C1—C2—C3—C8	1.3 (4)	C4—C3—C8—C7	−4.1 (4)
C1—C2—C3—C4	−176.1 (3)	C6—C7—C8—C9	−178.1 (3)
C8—C3—C4—C5	3.1 (4)	C11—C7—C8—C9	1.1 (5)
C2—C3—C4—C5	−179.6 (3)	C6—C7—C8—C3	1.2 (4)
C8—C3—C4—C12	−175.4 (3)	C11—C7—C8—C3	−179.5 (3)
C2—C3—C4—C12	2.0 (4)	C3—C8—C9—C10	1.6 (5)
C3—C4—C5—C6	0.8 (5)	C7—C8—C9—C10	−179.1 (3)
C12—C4—C5—C6	179.3 (3)	C8—C9—C10—C1	0.2 (5)
C4—C5—C6—C7	−3.9 (5)	C2—C1—C10—C9	−1.3 (5)
C5—C6—C7—C8	2.7 (5)	C5—C4—C12—O	146.1 (3)
C5—C6—C7—C11	−176.5 (3)	C3—C4—C12—O	−35.4 (5)
C2—C3—C8—C9	−2.3 (4)	C5—C4—C12—C13	−34.8 (4)
C4—C3—C8—C9	175.2 (3)	C3—C4—C12—C13	143.7 (3)

### Hydrogen-bond geometry (Å, °)

**Table:**

*D*—H···*A*	*D*—H	H···*A*	*D*···*A*	*D*—H···*A*
C2—H2A···O	0.93	2.30	2.920 (4)	124
C13—H13C···O^i^	0.96	2.55	3.296 (4)	135

Symmetry codes: (i) −*x*+3/2, *y*+1/2, *z*.

## References

[bb9] Allen, F. H., Kennard, O., Watson, D. G., Brammer, L., Orpen, A. G. & Taylor, R. (1987). *J. Chem. Soc. Perkin Trans. 2*, pp. S1–19.

[bb1] Dixon, E. A., Fischer, A. & Robinson, F. P. (1981). *Can. J. Chem* **59**, 2629–2641.

[bb2] Enraf–Nonius (1989). *CAD-4 Software* Enraf–Nonius, Delft, The Netherlands.

[bb3] Grummitt, O. & Buck, A. C. (1943). *J. Am. Chem. Soc* **65**, 295–296.

[bb4] Harms, K. & Wocadlo, S. (1995). *XCAD4* University of Marburg, Germany.

[bb5] Merritt, C. & Braun, C. E. (1950). *Org. Synth* **30**, 1–2.

[bb6] North, A. C. T., Phillips, D. C. & Mathews, F. S. (1968). *Acta Cryst.* A**24**, 351–359.

[bb7] Sheldrick, G. M. (2008). *Acta Cryst.* A**64**, 112–122. 10.1107/S0108767307043930 18156677

[bb8] Spek, A. L. (2003). *J. Appl. Cryst.* **36**, 7–13.

